# Comparison of flexible ureteroscopy with flexible and navigable suction ureteral access sheath and mini-percutaneous nephrolithotripsy for the treatment of impacted upper ureteral stones: a retrospective study

**DOI:** 10.3389/fsurg.2025.1562428

**Published:** 2025-03-27

**Authors:** Haiyang Tang, Yulong Che, Zhanpeng Wu, Fangchao Yuan, Jiayu Liu, Jie Li

**Affiliations:** Department of Urology, The First Affiliated Hospital of Chongqing Medical University, Chongqing, China

**Keywords:** flexible and navigable suction ureteral access sheath, flexible ureteroscopy, mini-percutaneous nephrolithotripsy, impacted stone, stone-free rate

## Abstract

**Background and objective:**

The treatment of impacted upper ureteral stones remains a significant challenge for urologists. Standard treatment protocols often favor Mini-Percutaneous Nephrolithotripsy (Mini-PCNL). It has been supposed to be associated with a higher stone clearance rate and a lower incidence of ureteral stricture compared to Flexible Ureteroscopy (FURS). Recently, FURS with flexible and navigable suction ureteral access sheath (FANS) has emerged as a promising alternative. The aim of this study was to compare the efficacy and safety of the FURS with FANS and Mini-PCNL for treating the impacted upper ureteral stones.

**Method:**

A retrospective study of 80 patients treated with FURS with FANS (Group A, *n* = 43) or Mini-PCNL (Group B, *n* = 37) was conducted in our center (from June 2023 to August 2024). Primary outcomes included stone-free rate (SFR), hemoglobin drop, hospital stay, and complications (Clavien-Dindo classification) in 3 months.

**Results:**

Both groups achieved comparable SFR (90.7% vs. 83.78%, *P* = 0.351). Group A had significantly lower hemoglobin drop (3.65 ± 8.39 vs. 7.89 ± 9.39 g/L, *P* = 0.036) and shorter hospital stays (1.79 ± 1.08 vs. 3.81 ± 1.37 days, *P* < 0.001). Complication rates were similar, but Group A had a higher rate of second-stage operation (18.6% vs. 8.1%, *P* = 0.174) and neither group required reoperation for ureteral stricture or rupture at 3 months post-surgery.

**Conclusion:**

FURS with FANS is a safe and effective alternative to Mini-PCNL for impacted upper ureteral stones larger than 10 mm, offering shorter recovery times and lower bleeding. However, its higher second-stage operation rate necessitates further investigation.

## 1 Introduction

The optimal surgical management for impacted upper ureteral stones continues to represent a therapeutic dilemma in contemporary endourological practice. Currently, there is no universally accepted definition of impacted upper ureteral stones. Generally, they are defined as stones that cause hydronephrosis, remain stationary for more than 4–8 weeks, and cannot be bypassed with contrast medium or a guidewire at the initial attempt (1, 2). Chronic irritation of the ureteral wall by impacted stones induces inflammatory cell proliferation and polyp formation. This pathological process results in ureteral distortion, subsequently increasing the risk of surgical complications including residual stones, ureteral perforation, and postoperative iatrogenic ureteral stricture. These factors collectively contribute to enhanced surgical complexity and associated risks (3).

Mini-Percutaneous Nephrolithotripsy (Mini-PCNL) and Flexible Ureteroscopy (FURS) are currently the most common treatment options for upper ureteral stones, both demonstrating excellent stone clearance and low recurrence rates (4, 5). FURS offers advantages of minimal invasiveness through natural passages and shorter hospital stays. However, in cases of impacted stones, patients face increased risks of surgical failure, ureteral rupture, and urosepsis. More seriously, ureteral stricture is a common complication after FURS, particularly in cases of impacted stones, with studies reporting up to a 24% chance of postoperative ureteral stricture (6). Mini-PCNL is easier to achieve a high stone free rate (SFR), but it is associated with higher bleeding risks and longer recovery times (7).

The ureteral sheath is a crucial instrument in FURS which provides a smooth tube in order to reduce friction and damage during the insertion and withdrawal of the ureteroscope. Pushing the ureteral stone back to the kidney for lithotripsy can help minimize ureteral damage and avoid postoperative ureteral stricture. However, it is difficult to clear all the stone fragment by FURS with conventional sheath especially in cases of impacted stones (8).

In recent years, the development of the flexible and navigable suction ureteral access sheath (FANS) has provided an effective solution to this problem. Compared to conventional sheaths, FANS demonstrates enhanced surgical efficacy through its flexible distal tip. It enables smoother navigation into steep infundibulum-pelvic angles (IPA) and facilitates effective stone fragment aspiration, although its effectiveness remains limited in certain lower calyces with steep IPA. It also simplifies and clarifies the assessment of the renal pelvis, ureter, and residual stone fragments after lithotripsy (9). The superiority of FURS with FANS surgery in the treatment of urinary stones has been consistently reported in the literature. Compared with the traditional sheath, FURS with FANS had a higher SFR and a lower risk of complications (10–14).

However, the comparative efficacy of Mini-PCNL and FURS with FANS in the treatment of impacted upper ureteral stones has not been adequately explored. In light of this, we compared retrospectively safety and efficiency of FURS with FANS and Mini-PCNL in treating impacted upper ureteral stones.

## 2 Patient and method

### 2.1 Inclusion criteria

This retrospective study included 80 patients with impacted upper ureteral stones diagnosed in the Urology Department of the First Affiliated Hospital of Chongqing Medical University between June 2023 and August 2024. All patients underwent treatment with either Mini-PCNL or FURS with FANS.

**Figure 1:**
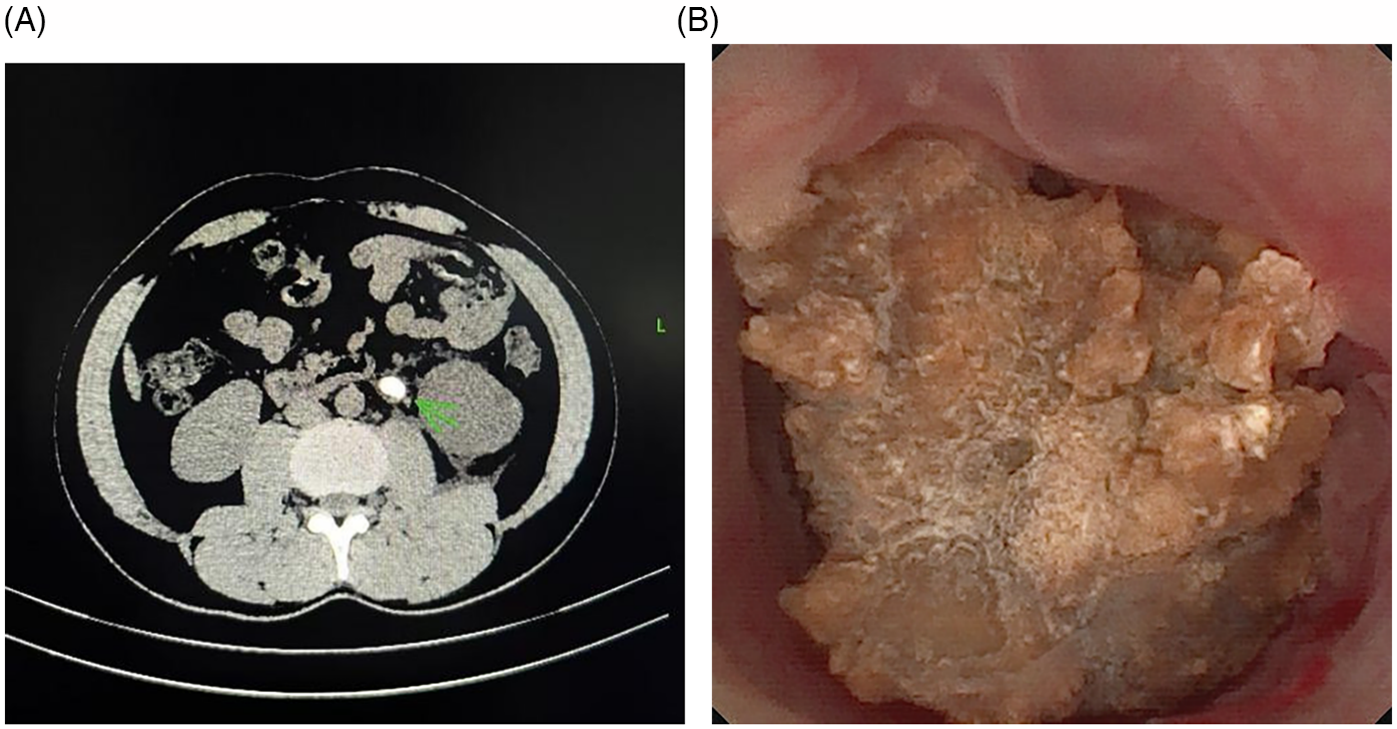
The impacted stone. **(A)** The stone impacted in the left ureter, ureteral wall thickening at the site of impaction and tissue edema surrounding the affected ureter. **(B)** The stone was observed to be tightly adherent to the ureteral wall in surgery. The mucosa at the site of impaction exhibited significant hyperemia and edema, with polyp formation noted.

1. Patients aged ≥18 years with stones measuring 1.0–2.0 cm in maximum diameter.
2. Stones located below ureteropelvic junction (UPJ) and above the lower border of the fourth lumbar vertebra.
3. The diagnosis of impacted upper ureteral stones was confirmed intraoperatively (Figure 1).

### 2.2 Exclusion criteria

1. Stones located in the middle or lower segments of the ureter.
2. Patients with combined kidney stones or contralateral urinary stones.
3. Uncontrolled infection, other systemic diseases that cannot tolerate surgery, and abnormal urinary tract anatomy.

### 2.3 Study protocol

Patients were divided into two groups based on the surgical method.

Group A: included 43 patients treated with FURS with FANS.
Group B: included 37 patients treated with Mini-PCNL.

All patients underwent complete history taking and physical examinations. Preoperative laboratory investigations included urine analysis, urine culture and routine blood tests. Urinary Tract CT and Abdominal Ultrasound were used as a preoperative standard to evaluate the size of the stone. The primary outcome was SFR (defined as zero fragment or a single fragment ≤2 mm) assessed using Kidney, Ureter, and Bladder x-ray (KUB) immediately after operation, followed by the ultrasound examination or CT scan 3 months postoperatively. The hemoglobin drop and postoperative hospital stay were recorded as indicators to evaluate the short-term surgical outcomes. All patients were followed for 3 months and complications were classified by the Clavien-Dindo classification.

### 2.4 Operative technique

**Figure 2:**
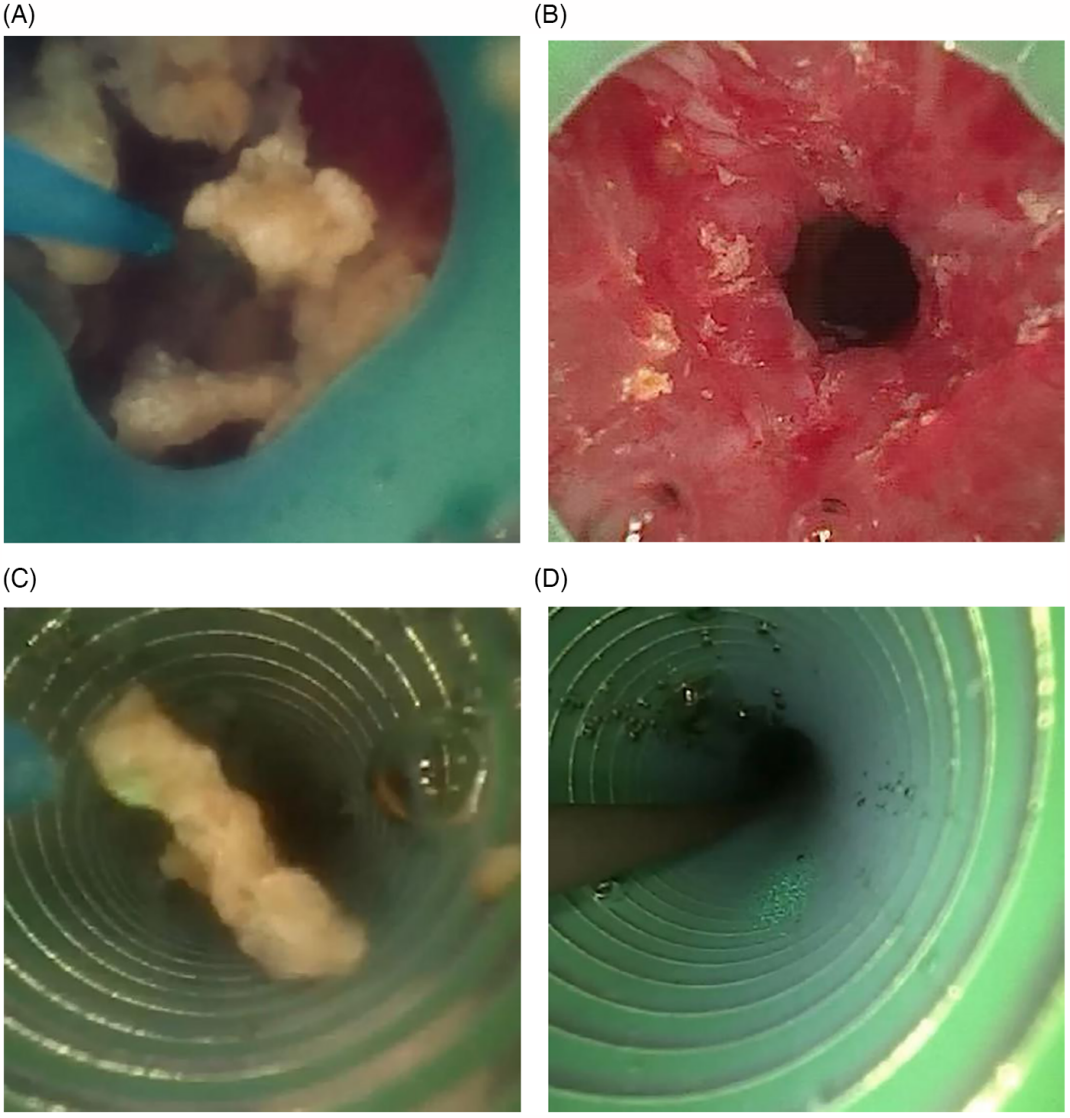
Treatment of impacted stones using FURS with FANS. **(A)** Pushing the stone back into the renal pelvis for lithotripsy due to protect ureter. **(B)** After pushing the stone back into the renal pelvis, ureteroscopic examination of the previous stone impaction site demonstrated no extensive iatrogenic ureteral trauma or structural disruption. **(C)** Utilizing FANS for fragment aspiration to prevent secondary ureteral impaction. **(D)** Placing stent under guidewire.

Group A (FURS with FANS): After general anesthesia, the patient was placed in the lithotomy position. A rigid ureteroscope was advanced into the ureter on the operative side to place a guidewire, and then a 11–13F FANS (Well Lead Medical, Guangzhou, China) was placed along the guidewire. A disposable flexible ureteroscope (Scivita Medical, Suzhou, China) was used to crush the stone with a holmium laser, attempting to return the stone to the kidney before or during lithotripsy to allow for further fragmentation and the sheath was used to suck the fragment under negative pressure. After the operation, a 5/6 F double-J stent (BOSTON TECH, USA) was inserted in the ureter on the operative side (Figure 2).

Group B (Mini-PCNL): Under general anesthesia, the patient was positioned in the prone position and the puncture needle was advanced into the pelvi-calyceal system under the guidance of ultrasound. A guidewire was placed through the inner stylet of the needle. Then, a 16/18F sheath (Well Lead Medical, Guangzhou, China) was placed along the guidewire at the puncture site to establish surgical access. After passing the access sheath with a nephroureteroscope, holmium laser was used for lithotripsy. Stone fragments were evacuated via a continuous irrigation system consisting of the 16/18F sheath and irrigation pump. After lithotripsy, a 5/6F double J stent (BOSTON TECH, USA) was inserted in the ureter on the operative side, and a percutaneous nephrostomy tube was placed.

### 2.5 Statistical analysis

The Statistical Package for the Social Sciences (SPSS, IBM, Chicago, IL, USA) was used for data analysis. *p*-value < 0.05 was considered statistically significant. The Student's *t*-test and the Mann–Whitney *U* test were used to assess the statistical significance of differences in parametric variables between the two study groups. Fisher's exact test and Pearson's Chi-squared Test were used to evaluate the association between two qualitative variables. Descriptive statistics were presented as mean ± standard deviation (SD) or numbers and percentages.

## 3 Ethical approval

This study was approved by the Ethics Committee of Chongqing Medical University. All patients' information was kept strictly confidential.

## 4 Result

The patient and stone characteristics are summarized in Table 1. The mean age of patients was 53.39 ± 12.56 years in Group A and 55.11 ± 15.74 years in Group B. In Group A, there were 36 male patients (83.72%) and 7 female patients (16.28%), while Group B included 29 male patients (78.38%) and 8 female patients (21.62%). The Body Mass Index (BMI) was 25.57 ± 3.11 kg/m² in Group A and 24.75 ± 2.67 kg/m² in Group B. There were no significant differences in sex, age and BMI between the two groups. The mean stone size was 12.94 ± 2.29 mm in Group A and 14.09 ± 3.11 mm in Group B. Similarly, no significant difference between two groups. Preoperative and postoperative data are presented in Table 2. Group A had comparable SFR with Group B (90.7% vs. 83.78%, *P* = 0.351). Group B had worse postoperative decreases in hemoglobin than Group A (7.89 ± 9.39 vs. 3.65 ± 8.39 g/L, *P* = 0.036) and the difference was statistically significant. Group A had a shorter postoperative hospital stay compared to Group B (1.79 ± 1.08 vs. 3.81 ± 1.37 days, *P* < 0.001). There was a higher rate of second-stage operation in Group A due to ureterostenosis and main reason for Group B was infection (18.60% vs. 8.11%, *P* = 0.174). In addition, there was no difference between the two groups in operative time, preoperative and postoperative inflammatory indicators. According to the Clavien-Dindo classification, 59 individuals were classified as grade Ⅰ (32 in Group A and 27 in Group B), while 21 cases at grade Ⅱ (11 in Group A and 10 in Group B). Neither group reported cases of postoperative ureteral stricture or rupture (Table 3).

**Table 1:** Patient characteristics at baseline.

Variable	Group A	Group B	t/z/x2	*P*-value
	(*n* = 43)	(*n* = 37)		
Gender (%)				
Male	36 (83.72)	29 (78.38)	0.373	0.542
Female	7 (16.28)	8 (21.62)		
Age (years)	53.39 ± 12.56	55.11 ± 15.74	−0.541	0.59
Body mass index (BMI, kg/m^2^)	25.57 ± 3.11	24.75 ± 2.67	1.239	0.219
Stone size (mm)	12.94 ± 2.29	14.09 ± 3.11	−1.909	0.06
History of stone surgery (%)				
Yes	10 (23.26)	10 (27.03)	0.151	0.698
No	33 (76.74)	27 (72.97)		
History of diabetes (%)				
Yes	8 (18.60)	9 (24.32)	0.389	0.533
No	35 (81.40)	28 (75.68)		
Laterality of stone (%)				
Left	24 (55.81)	24 (64.86)	0.679	0.41
Right	19 (44.19)	13 (35.14)		
Location of stone (%)				
Upper	36 (83.72)	28 (75.68)	0.805	0.37
Ureteropelvic junction (UPJ)	7 (16.28)	9 (24.32)		
Perirenal fluid stranding (%)				
Yes	19 (44.19)	14 (37.84)	0.331	0.565
No	24 (55.81)	23 (62.16)		

**Table 2:** Operative characteristics.

Variable	Group A	Group B	t/z/x2	*P*-value
	(*n* = 43)	(*n* = 37)		
Operation time (minutes, min)	59.74 ± 28.29	59.65 ± 31.49	0.014	0.989
White blood cells (10^9^/L)				
Preoperative	6.76 (5.40–8.13)	6.28 (4.99–8.22)	−0.444	0.657
Postoperative	9.20 (6.84–11.04)	8.91 (6.75–11.47)	−0.145	0.885
Urine white blood cells (cells)				
Preoperative	48.00 (26.00–148.00)	70.00 (30.00–160.50)	−0.825	0.409
Postoperative	88.00 (54.00–143.00)	52.00 (18.50–125.00)	−0.767	0.443
Hemoglobin drop (ΔHb, g/L)	3.65 ± 8.39	7.89 ± 9.39	−2.134	0.036
Stone clearance rate (SFR, %)	90.7	83.78	0.869	0.351
Second-stage operation (%)				
Yes	8 (18.60)	3 (8.11)	1.848	0.174
No	35 (81.40)	34 (91.89)		
Postoperative hospital stay (days)	1.79 ± 1.08	3.81 ± 1.37	−7.362	<0.001

This illustrates the comparable SFR between group A and group B (90.7% vs. 83.78%, *P* = 0.351). Despite similar operative times, Group A demonstrated significantly lower hemoglobin drop (*P* = 0.036) and shorter hospital stays (*P* < 0.001), indicating a faster recovery process.

**Table 3:** The complication in 3 months.

Grade	Complication	Group A (%)	Group B (%)
Grade I	Postoperative hematuria, pain and fever not requiring antibiotic treatment	66.67% (32)	80.95% (27)
Grade II	Urinary tract infection (UTI) requiring antibiotics	33.33% (11)	19.05% (10)
Grade III	Postoperative ureterostenosis or rupture	0	0
Grade IV	Organ dysfunction and urosepsis	0	0
Grade V	Postoperative death	0	0

This illustrates that according to Clavien-Dindo classification, both groups reported similar condition and no one required reoperation for ureterostenosis or rupture.

## 5 Discussion

In this study, we retrospectively evaluated the safety and efficacy of FURS with FANS and Mini-PCNL in the management of impacted upper ureteral stones.

The present study demonstrated that FURS with FANS was an effective approach for the treatment of impacted upper ureteral stones. Mohey A et al. conducted a prospective study to evaluate the efficacy of conventional sheaths with FURS and PCNL for impacted ureteral stones (15). Their findings suggested that PCNL had superior SFR compared to FURS. However, our study found that FURS with FANS could achieve an equally effective SFR compared to Mini-PCNL. The impacted stone usually causes edema, erosion, and polyp growth of the ureteral mucosa, which will interfere with stone removal. To minimize this risk, stones were pushed back into the kidney before lithotripsy. However, due to the same pathological changes, the discharge of stone fragments postoperatively remains challenging. The flexible tip design of FANS enables access to renal calyces that conventional sheaths cannot reach, facilitating efficient stone fragment aspiration. This feature improves SFR and prevents fragment re-impaction in narrow anatomical segments (16, 17).

Similarly, the effectiveness of FANS is still limited by ureteral conditions. In cases of ureterostenosis or severe infection, a second-stage operation will be required, increasing hospitalization time and the risk of infection or thrombosis (18). In this study, eight cases in Group A underwent second-stage operation for stenosis, compared to only three cases in Group B, which were attributed to infection. This study found no difference in operative time between FURS with FANS and Mini-PCNL (59.74 ± 28.29 vs. 59.65 ± 31.49, *P* = 0.989). Previous studies on differences in operative time between FURS and PCNL had been inconclusive. On the one hand, Mini-PCNL may require a longer time due to the establishment of the PCNL tract. However, on the other hand, it is more effective than FURS in terms of stone fragmentation efficiency (19, 20). Zhu et al. reported that the operative time of PCNL was shorter than FURS with FANS for the treatment of renal or ureteral stones ≤30 mm (10). This difference may be attributed to the repeated insertion and withdrawal of the scope through FANS during stone suction (21).

Postoperative complications most commonly include bleeding and infection. Mini-PCNL, due to its invasive nature, carries a higher risk of intraoperative bleeding and blood transfusion compared to FURS, which is one of the reasons for selecting FURS (22). In this study, the extent of hemoglobin drop was employed as an indicator to evaluate intraoperative bleeding. Notably, there was significant difference in hemoglobin drop between the two groups. The Mini-PCNL group showed a higher mean value than the FURS group (7.89 ± 9.39 vs. 3.65 ± 8.39 g/L, *P* = 0.036), suggesting that FURS may offer a superior safety profile for patients with anemia or coagulation disorders. According to the Clavien-Dindo score, most complications in both groups were classified as Grade I or II. Furthermore, At 3 months follow-up, neither group required reoperation for ureterostenosis or rupture. It was suggested that pushing ureteral stones back into the kidney for lithotripsy under FURS with FANS and aspirating the fragments using negative pressure can avoid postoperative ureteral stricture while achieving a stone clearance rate comparable to Mini-PCNL.

A significant discrepancy was observed in the postoperative hospitalization duration between the two groups. Group A demonstrated shorter hospital stays compared to Group B (1.79 ± 1.08 vs. 3.81 ± 1.37 days, *P* < 0.001). Cheng et al. reported that FURS caused minimal postoperative pain or bleeding, had less impact on hemoglobin levels and coagulation, and offered advantages such as reduced bleeding, shorter hospital stays, and faster gastrointestinal recovery (23). Considering the invasive nature of Mini-PCNL and the potential of patients having a long-term indwelling nephrostomy tube after surgery, it can be argued that at the same SFR, FURS with FANS enhanced postoperative comfort, reduced nursing workload and delivered superior short-term surgical outcomes.

## 6 Conclusion

FURS with FANS for the management of impacted upper ureteral stones is a safe and feasible procedure, offering a high SFR, minimal bleeding, and shorter hospital stays, reducing the risk of postoperative ureteral stricture. However, compared to Mini-PCNL, it has a higher rate of second-stage operation due to ureteral conditions.

## 7 Limitation

Further studies are required to validate the findings comparing FURS with FANS and Mini-PCNL. The long-term efficacy and safety of these two surgical approaches require further investigation to better inform clinical practice.

## Data Availability

The original contributions presented in the study are included in the article/Supplementary Material, further inquiries can be directed to the corresponding author.
